# Integrative analysis of competing endogenous RNA network focusing on long noncoding RNA associated with progression of cutaneous melanoma

**DOI:** 10.1002/cam4.1315

**Published:** 2018-03-09

**Authors:** Siyi Xu, Jing Sui, Sheng Yang, Yufeng Liu, Yan Wang, Geyu Liang

**Affiliations:** ^1^ Key Laboratory of Environmental Medicine Engineering Ministry of Education School of Public Health Southeast University Nanjing Jiangsu China; ^2^ TCM of Jiangsu Provincial Hospital Nanjing Jiangsu China; ^3^ Institute of Dermatology Chinese Academy of Medical Sciences and Peking Union Medical College Jiangsu Key Laboratory of Molecular Biology for Skin Diseases and STIs Nanjing Jiangsu China

**Keywords:** ceRNA network, cutaneous melanoma, lncRNA, progression

## Abstract

Cutaneous melanoma (CM) is the most malignant tumor of skin cancers because of its rapid development and high mortality rate. Long noncoding RNAs (lncRNAs), which play essential roles in the tumorigenesis and metastasis of CM and interplay with microRNAs (miRNAs) and mRNAs, are hopefully considered to be efficient biomarkers to detect deterioration during the progression of CM to improve the prognosis. Bioinformatics analysis was fully applied to predict the vital lncRNAs and the associated miRNAs and mRNAs, which eventually constructed the competing endogenous RNA (ceRNA) network to explain the RNA expression patterns in the progression of CM. Further statistical analysis emphasized the importance of these key genes, which were statistically significantly related to one or few clinical features from the ceRNA network. The results showed the lncRNAs MGC12926 and LINC00937 were verified to be strongly connected with the prognosis of CM patients.

## Introduction

Cutaneous melanoma (CM), a malignant tumor developing from melanocytes, is considered to be the most aggressive of skin cancers. Although the number of CM cases only accounts for less than 3% of skin cancer cases, its incidence has been on the rise for the past 30 years, with a faster rate than any other type of skin cancer worldwide 1, 2. In addition, it results in more than 70% of patient deaths, most of which were patients in stages III and IV of the disease. It is concluded that patients with early‐stage melanoma are considerably cured, while in the later stages (stages III and IV), the thickness of the tumor (more than 2 mm) and distant metastasis are regarded as the greatest risk factors 3, 4, 5, 6.

Despite the early diagnosis of CM to efficiently improve the prognosis of patients, to date, there is no undisputable detection of this cancer. The morphological diagnosis of CM is based on imaging techniques, such as MRI and CT, which lack sensitivity to distinguish early lesions. In addition, histopathological interpretation cannot completely diagnose CM 7, 8. As a result, the 5‐year survival rate is poor, at less than 16% 9. Obviously, a sensitive and specific biomarker that can distinguish early lesions of CM will make a tremendous contribution to diagnosis and prognosis as well as mitigate the social burden of this disease.

Long noncoding RNAs (lncRNAs) are one of the transcripts without a coding protein having 200–10 000 bp in length. They interact in a regulatory manner before, during, and after transcription. The multiple levels of regulation in lncRNA exhibit huge potential in diverse biological processes, of which cell proliferation, differentiation, and migration are closely associated with metastasis and the deterioration of tumors 10, 11. In addition, lncRNAs display a tissue‐specific expression pattern 12. Many studies have confirmed the participation of various lncRNAs in the etiology and carcinomatosis of CM 13, 14, 15, 16. Therefore, we examined the expression of different lncRNAs between the stages of CM to detect whether one or more lncRNAs could serve as biomarkers.

## Materials and Methods

### TCGA database and bioinformatics analysis

**Figure 1:**
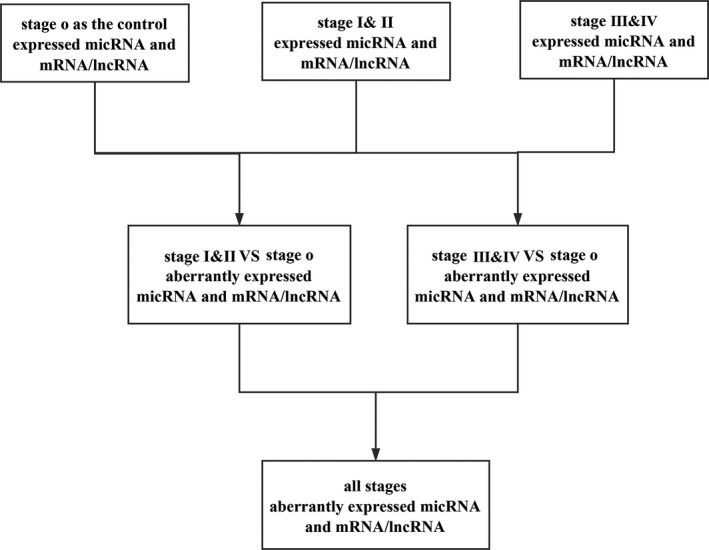
The flowchart of the bioinformatics analysis.

The RNA expression data (level 3: which were processed and standardized based on the miRNA expression data of TCGA.) and clinical information of CM patients were downloaded from The Cancer Genome Atlas Data Portal as of December 2016. All the original RNA sequencing raw reads were processed and normalized by the TCGA RNASeqV2 system afterward to fit the analysis. To seek the relationship between the RNA expression and the metastasis of melanoma, all the patients in TCGA were divided into three groups (stage 0, stages I & II, and stages III & IV) according to their diagnosed stages based on AJCC (the American Joint Committee on Cancer). Because of the lack of data, the numbers of groups varied between microRNAs and others. There were 6207 and 179 patients in three microRNA (miRNA) groups, respectively, while there were 6220 and 182 patients who composed another three groups of mRNA/lncRNAs samples. For the purpose of pointing out a cluster of lncRNA, mRNA, and miRNA, the bioinformatics analysis was conducted following the procedures in the flowchart of Figure 1.

### Functional enrichment analysis

To understand the biological functions and processes these genes were involved in, Gene Ontology database (GO, http://www.geneontology.org) and Kyoto Encyclopedia of Genes and Genomes (KEGG, http://www.kegg.jp/) were utilized to conduct functional enrichment analysis. The enrichment score was regarded above 1.5 or below 0.67 with *P* < 0.05.

### Construction of the competing endogenous RNA (ceRNA) and protein–protein interaction (PPI) networks

**Figure 2:**
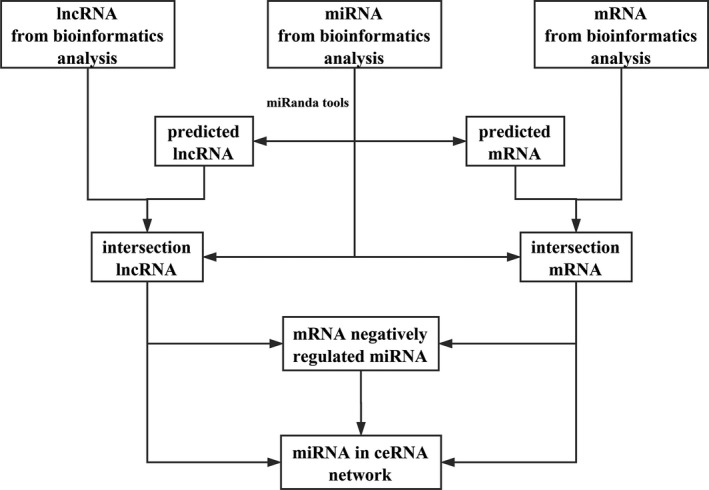
The flowchart of the ceRNA network construction.

With the relationship among lncRNAs, miRNAs, and mRNAs, we can predict related sponge lncRNAs through miRNAs based on the MREs (miRNA Response Elements) and predict mRNAs through the miRNAs that invoke their expression by binding them 17. The miRanda tool (http://www.microrna.org/microrna/home.do) was performed to provide the lncRNA–miRNA and miRNA–mRNA interactions, and all the results can be verified through starBase V2.0 database (http://starbase.sysu.edu.cn). These predicted results were cross‐matched with the results of the bioinformatics analysis. Finally, the mRNAs with no negatively regulated lncRNAs and miRNAs were discarded. Hence, a map of the interplay among lncRNAs, miRNAs, and mRNAs based on the previous bioinformatics analysis was completed. Only the lncRNAs, miRNAs, and mRNAs with fold changes >1.5/<0.67 and *P* < 0.05 were retained. The designs and details are presented in the flowchart of Figure 2. The visualization of the ceRNA network was accomplished by Cytoscape v3.0. Afterward, in context with the co‐expressed genes, the PPI network was conducted via STRING (Version 10.5) (https://string-db.org/).

### Statistical analysis of key genes and clinical features

Key genes closely associated with the metastasis of CM were filtrated by the bioinformatics analysis and the ceRNA network, successively. To master more information about how these key genes affect CM, they were deeply analyzed according to clinical features, including gender, race, AJCC pathological stage, TNM stage, and outcome with Student's *t*‐test. In addition, Kaplan–Meier survival curves were set up to identify the specific lncRNAs associated with CM patients' survival time. All results with *P* < 0.05 were considered to be statistically significant. The statistical analyses were performed using SPSS 23.0.

## Results

### Specific lncRNAs in the progression of CM

**Figure 3:**
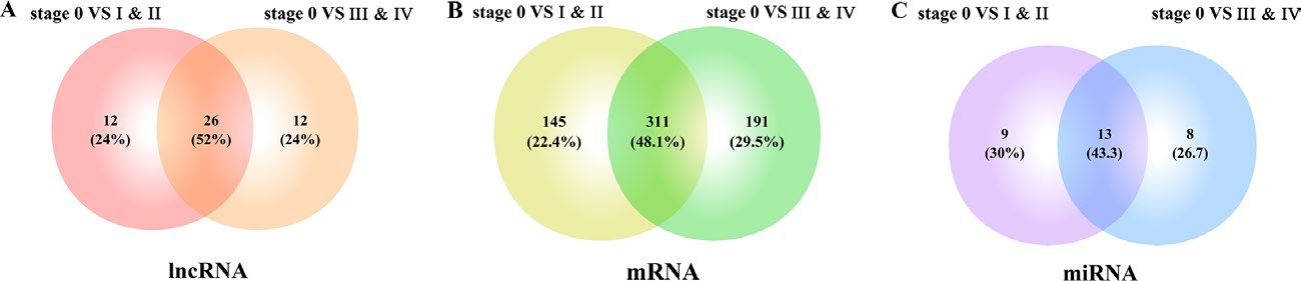
Venn diagram analysis of aberrantly expressed (A) lncRNA, (B) mRNA, (C) miRNA between CM patients of stages I & II and III & IV with stage 0.

Through bioinformatics analysis, specific expressed lncRNAs, mRNAs, and miRNAs at different stages were identified with the standard of absolute fold changes >1.5 or <0.67 and *P* < 0.05. Comparing the expression of lncRNAs in stages I & II of CM with those in stage 0, 38 aberrantly expressed lncRNAs were identified, and another 38 lncRNAs were screened out through the same comparison between stages III & IV and stage 0. After cross‐matching the results, 26 lncRNAs, which were aberrantly expressed throughout the development of CM, were identified (Fig. 3, Table 1). The comparison of the expression of mRNAs between other stages with stage 0 resulted in 456 mRNAs that were aberrantly expressed in the early stages and 502 mRNAs in the advanced stages, and there were 311 mRNAs included in both levels (Fig. 3). However, after the same process, only 13 miRNAs, which were aberrantly expressed between different stages with stage 0, were unearthed (Fig. 3).

**Table 1:** Aberrantly expressed intersection of lncRNAs between stages I & II versus stage 0 and stages III & IV versus stage 0

lncRNAs	Gene ID	Regulation	Fold change (stages I & II/0)	*P*‐value^a^ (stages I & II/0)	FDR (stages I & II/0)	Fold change (stages III & IV/0)	*P*‐value^b^ (stages III & IV/0)	FDR (stages III & IV/0)
LPAL2	80,350	Down	0.28	<0.05	0.999	0.22	<0.01	1
MGC12916	84,815	Down	0.29	<0.01	0.999	0.32	<0.05	1
ATP1A1‐AS1	84,852	Down	0.54	<0.05	0.999	0.53	<0.05	1
LOC90768	90,768	Down	0.15	<0.05	0.999	0.20	<0.05	1
LINC01341	149,134	Down	0.29	<0.05	0.999	0.35	<0.05	1
LOC150776	150,776	Down	0.65	<0.05	0.999	0.61	<0.05	1
DNM1P46	196,968	Down	0.33	<0.05	0.999	0.29	<0.01	1
CYP4F35P	284,233	Down	0.34	<0.05	0.999	0.28	<0.01	1
LINC00965	340,196	Down	0.52	<0.05	0.999	0.54	<0.05	1
MRPL42P5	359,821	Down	0.42	<0.05	0.999	0.39	<0.05	1
RPL13AP20	387,841	Down	0.57	<0.05	0.999	0.57	<0.05	1
LOC400794	400,794	Down	0.39	<0.05	0.999	0.39	<0.05	1
LOC401127	401,127	Down	0.50	<0.05	0.999	0.44	<0.01	1
TTLL13P	440,307	Down	0.45	<0.05	0.999	0.43	<0.01	1
LOC440461	440,461	Down	0.50	<0.05	0.999	0.46	<0.01	1
SEC1P	653,677	Down	0.49	<0.05	0.999	0.42	<0.01	1
SRRM2‐AS1	100,128,788	Down	0.53	<0.05	0.999	0.45	<0.01	1
TEKT4P2	100,132,288	Down	0.45	<0.05	0.999	0.42	<0.01	1
TP73‐AS1	57,212	Up	3.06	<0.01	0.999	2.67	<0.05	1
LINC01102	150,568	Up	5.65	<0.05	0.999	4.60	<0.05	1
LINC00937	389,634	Up	2.22	<0.05	0.999	2.37	<0.05	1
ZNF321P	399,669	Up	2.39	<0.05	0.999	2.48	<0.05	1
HCG11	493,812	Up	3.05	<0.05	0.999	3.26	<0.01	1
RPL23AP53	644,128	Up	2.20	<0.01	0.868	2.15	<0.01	1
LOC646762	646,762	Up	2.49	<0.01	0.899	2.43	<0.01	1
CDKN2B‐AS1	100,048,912	Up	3.48	<0.05	0.999	3.53	<0.05	1

a Derived from the comparison between patients of stages I & II and stage 0.

b Derived from the comparison between patients of stages III & IV and stage 0.

### GO enrichment and KEGG pathway analysis

**Figure 4:**
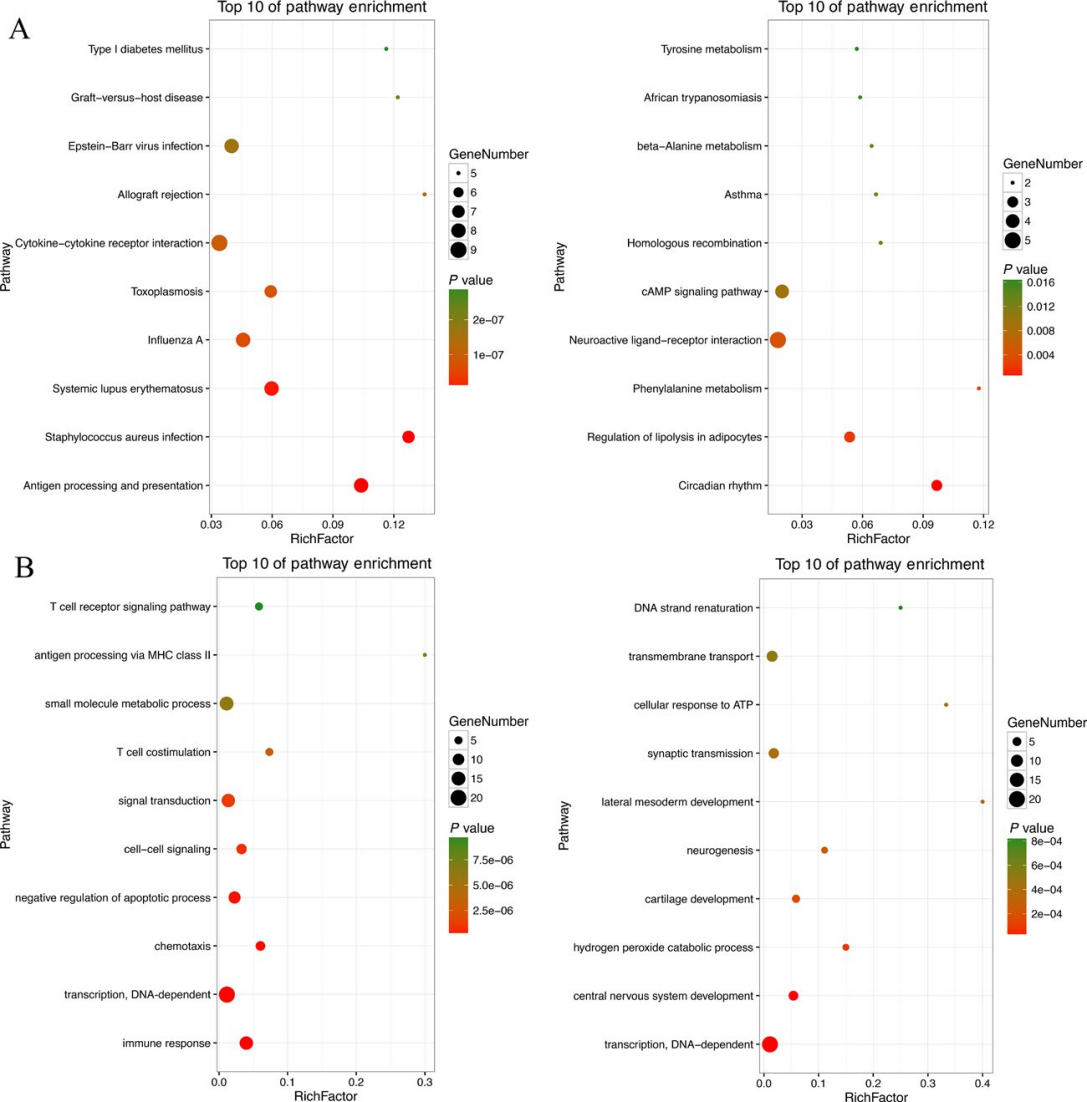
Top 10 enrichment of pathways (A) and GO (B) analysis for aberrantly expressed intersection of mRNAs.

To gather more information about the molecular functions and signal pathways of the genes that had been selected, GO and KEGG were utilized to analyze the upregulated and downregulated genes, separately. The record with *P*‐value <0.05 and enrichment >2.0 was preserved. It revealed 223 GO processes of upregulated genes and 147 downregulated genes. In addition, KEGG found 41 categories corresponded to upregulated genes and 14 categories corresponded to downregulated genes. The top 10 of both upregulated and downregulated pathway enrichments are described in Figure 4.

Among the top 10 upregulated pathways, over half of them were related to immunization directly and indirectly, and the pathway of asthma had its presence among the top 10 downregulated pathways, where a vital pathway was identified—the cAMP signaling pathway. Extensively known, the cAMP signaling pathway is involved in many living activities and biological process, mainly through its deep effect on cell motility, proliferation, and apoptosis, which are remarkably associated with the genesis of carcinoma 18, 19, 20. In addition, three pathways concerning the metabolism of three amino acids, including phenylalanine, tyrosine, and beta‐alanine, were noted. Phenylalanine is one of the eight essential amino acids that cannot be synthesized de novo in the human body and other living beings but works after being transformed into tyrosine by phenylalanine hydroxylase. In succession, as a raw material, tyrosine can be converted to melanin in melanocytes.

The top 10 upregulated and downregulated GO terms are also identified in Figure 4. In detail, most upregulated genes took part in communication and signal transduction of cells, especially T cells, while downregulated genes participated in the development of multiple systems. Collectively, GO enrichment analysis indicated immunization in the metastasis of CM and that the top upregulated enriched GO term was the immune response.

### ceRNA and PPI network

**Figure 5:**
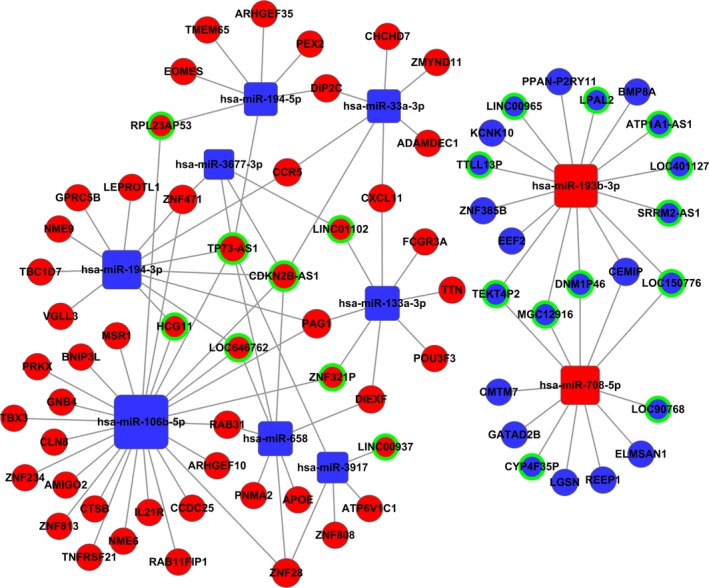
Competing endogenous network. Squares represent miRNAs, balls represent mRNAs, and balls with a green circle around represents lncRNAs. Red means upregulated genes, while blue means downregulated genes.

**Figure 6:**
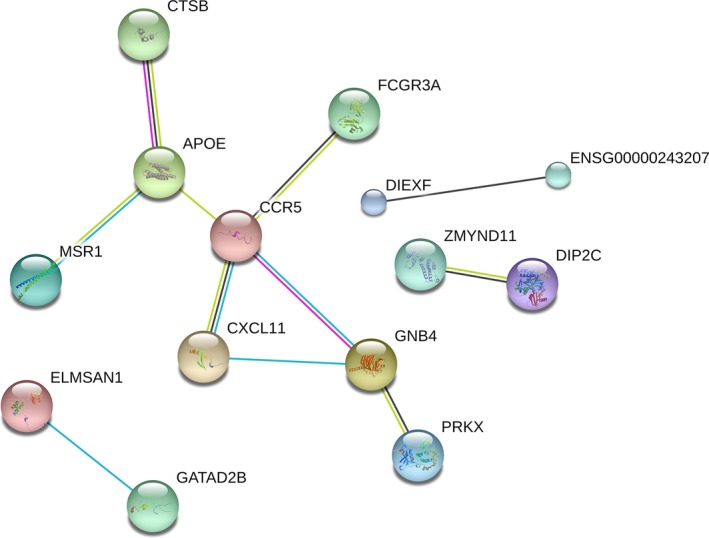
The protein–protein interaction network constructed for the aberrantly expressed genes.

To set up the lncRNA–miRNA–mRNA ceRNA network, first the 13 aberrantly expressed miRNAs and 26 specific lncRNAs were utilized to explore the targeted relationship with the utilization of miRanda tools and starBase V2.0 database. With this, 23 lncRNAs, which have specific MREs, had a regulated relationship with 13 miRNAs (Table 2). Next, mRNAs targeted by miRNAs were uncovered based on these 13 miRNAs and the 311 mRNAs, which were preliminarily considered associated with the deterioration of CM. Afterward, 122 mRNAs were left in the list (Table 3). However, three (hsa‐miR‐192‐5p, hsa‐miR‐194‐5p, and hsa‐miR‐891a‐5p) of the thirteen miRNAs did not have related negatively regulated lncRNAs. Finally, the numbers of key lncRNAs, miRNAs, and mRNAs reduced to 20, 10, and 54, respectively, which constituted the ceRNA network (Fig. 5, and the alignments between genes are presented in Table S1 and S2). Moreover, the PPI network furnished 106 genes, which were regarded as central genes (Fig. 6).

**Table 2:** miRNAs and specific targeted intersection of key lncRNAs associated with the development of CM

Key lncRNAs	miRNAs
ATP1A1‐AS1	hsa‐miR‐106b‐5p,hsa‐miR‐133a‐3p,hsa‐miR‐193b‐3p,hsa‐miR‐194‐3p
CDKN2B‐AS1	hsa‐miR‐106b‐5p,hsa‐miR‐194‐3p,hsa‐miR‐33a‐3p,hsa‐miR‐3677‐3p,hsa‐miR‐658,hsa‐miR‐708‐5p
CYP4F35P	hsa‐miR‐33a‐3p, hsa‐miR‐708‐5p
DNM1P46	hsa‐miR‐1‐3p, hsa‐miR‐106b‐5p, hsa‐miR‐133a‐3p, hsa‐miR‐193b‐3p, hsa‐miR‐658, hsa‐miR‐708‐5p
HCG11	hsa‐miR‐106b‐5p, hsa‐miR‐194‐3p
LINC00937	hsa‐miR‐3917
LINC00965	hsa‐miR‐193b‐3p, hsa‐miR‐194‐3p, hsa‐miR‐658
LINC01102	hsa‐miR‐133a‐3p, hsa‐miR‐3677‐3p
LINC01341	hsa‐miR‐3677‐3p
LOC150776	hsa‐miR‐193b‐3p, hsa‐miR‐194‐3p, hsa‐miR‐3677‐3p, hsa‐miR‐658, hsa‐miR‐708‐5p
LOC400794	hsa‐miR‐133a‐3p, hsa‐miR‐658
LOC401127	hsa‐miR‐193b‐3p, hsa‐miR‐658
LOC646762	hsa‐miR‐106b‐5p, hsa‐miR‐194‐3p, hsa‐miR‐658, hsa‐miR‐708‐5p
LOC90768	hsa‐miR‐1‐3p, hsa‐miR‐192‐5p, hsa‐miR‐133a‐3p, hsa‐miR‐194‐3p, hsa‐miR‐3677‐3p, hsa‐miR‐3917, hsa‐miR‐658, hsa‐miR‐708‐5p
LPAL2	hsa‐miR‐193b‐3p, hsa‐miR‐194‐3p
MGC12916	hsa‐miR‐193b‐3p, hsa‐miR‐194‐3p, hsa‐miR‐33a‐3p, hsa‐miR‐658, hsa‐miR‐708‐5p
MRPL42P5	hsa‐miR‐1‐3p, hsa‐miR‐33a‐3p, hsa‐miR‐658
RPL13AP20	hsa‐miR‐3677‐3p, hsa‐miR‐658
RPL23AP53	hsa‐miR‐106b‐5p, hsa‐miR‐194‐5p,hsa‐miR‐708‐5p,hsa‐miR‐891a‐5p
SEC1P	hsa‐miR‐3917,hsa‐miR‐658
SRRM2‐AS1	hsa‐miR‐193b‐3p,hsa‐miR‐3677‐3p
TEKT4P2	hsa‐miR‐193b‐3p,hsa‐miR‐194‐3p,hsa‐miR‐194‐5p,hsa‐miR‐3677‐3p,hsa‐miR‐658,hsa‐miR‐708‐5p
TP73‐AS1	hsa‐miR‐106b‐5p,hsa‐miR‐193b‐3p,hsa‐miR‐194‐3p,hsa‐miR‐194‐5p,hsa‐miR‐3677‐3p, hsa‐miR‐3917, hsa‐miR‐658
TTLL13P	hsa‐miR‐193b‐3p
ZNF321P	hsa‐miR‐106b‐5p,hsa‐miR‐133a‐3p,hsa‐miR‐708‐5p

**Table 3:** miRNAs and specific targeted mRNAs in the progression of CM

miRNAs	mRNAs
hsa‐miR‐106b‐5p	AMIGO2, ARHGEF10, ATP2B2, BNIP3L, CC2D1A, CCDC25, CLN8, CNNM3, CTSB, DIS3L, EPX, FBXO39, FKRP, GNB4, HSD17B1, IL21R, IL27RA, KCNK10, MSR1, NME6, PAG1, PER2, PRKX, RAB11FIP1, RAB31, RPS6KA2, STK33, TBX3, TNFRSF21, VWA2, ZNF234, ZNF28, ZNF471, ZNF813
hsa‐miR‐133a‐3p	CXCL11, DDIT4, DIEXF, DLGAP1, FAM57B, FCGR3A, PAG1, POU3F3, TTN
hsa‐miR‐1‐3p	ASH2L, BMPER, COL25A1, CYFIP2, DIP2C, EPHB1, HLA‐DQA1, INHBA, KCTD16, RGS5, SH3BP5, TNS3
hsa‐miR‐192‐5p	ATP6V1C1, BMPER, C8orf46, CDS1, DDHD2, GOLGA6B, MKNK2, UMODL1, VWA2
hsa‐miR‐193b‐3p	BMP8A, CEMIP, CTSC, EEF2, EOMES, KCNK10, PAG1, PPAN‐P2RY11ZNF385B
hsa‐miR‐194‐3p	ADM5, ATP2B2, ATXN7L2, BMP8A, CCR5, CNNM3, CNTN2, CRTC1, DIO3, DOK7, FKRP, GATAD2B, GPRC5B, KIAA0556, LEPROTL1, LYVE1, NHLH1, NME9, NOXA1, PAG1, PDK2, REEP1, RPL28, SIPA1L3, TBC1D7, TREML1, VGLL3, ZNF471
hsa‐miR‐194‐5p	ARHGEF35, DDX11, DIP2C, DMRT2, EOMES, PEX2, SFMBT1, TENM3, TMEM65
hsa‐miR‐33a‐3p	ADAMDEC1, CCR5, CEMIP, CHCHD7, CHRNA3, CXCL11, DIO3, DIP2C, DLGAP1, DMRTC1B, ZMYND11
hsa‐miR‐3677‐3p	CNTN2, DOK7, SLC18A1, VIPR2, ZMIZ1, ZNF471
hsa‐miR‐3917	ATP6V1C1, CRTC1, IL27RA, SFMBT1, ZNF28, ZNF808
hsa‐miR‐658	APOE, CPNE9, DDX11, DIEXF, EBF2, ELMSAN1, GABRR1, HOXA7, MKNK2, OTP, PDK2, PLIN4, PNMA2, RAB31, SLC18A1, TUSC5, ZMIZ1, ZNF28, ZNF609
hsa‐miR‐708‐5p	CCDC25, CEMIP, CMTM7, CTSC, CXCL9, DIEXF, ELMSAN1, GATAD2B, LGSN, REEP1, SMIM19, TNS3, TPMT, ZMYND11, ZNF234
hsa‐miR‐891a‐5p	KIAA0556

### Association between key genes and clinical features

Now that the ceRNA network provided vast potential genes that consisted of 20 lncRNAs, 10 miRNAs, and 54 mRNAs, the association between these genes and the clinical features can be assessed to preliminarily reveal to what degree and in which aspect these genes play a role in the progression of CM.

The results of lncRNAs are shown in Table 4, suggesting that 14 lncRNAs were markedly associated with the CM TNM stages, tumor metastasis, and the patients' outcome. Unfortunately, none of the lncRNAs in the ceRNA network were positive in the comparisons of other clinical features such as gender, race and pathological stage. Notably, SRRM2‐AS1, ATP1A1‐AS1 and RPL23AP53 were involved in TNM stages and tumor metastasis. In addition, LOC401127, which has a connection to tumor metastasis, was involved in the outcome of patients as well.

**Table 4:** The correlation between specific lncRNAs from the ceRNA network and clinical features of CM patients

Clinical features	Upregulated lncRNAs	Downregulated lncRNAs
Gender (Female vs. Male)		
Race (White vs. Asian)		
Tumor pathological stage (III & IV vs. I & II)		
TNM staging system (T3 + T4 vs. T1 + T2)	ATP1A1‐AS1, RPL23AP53, SRRM2‐AS1, DNM1P46	
Tumor metastasis (primary solid tumor vs. metastatic tumor)	LINC00965, LOC401127	LOC150776, TP73‐AS1, LPAL2, SRRM2‐AS1, ATP1A1‐AS1, LOC646762, RPL23AP53, LINC01102, TEKT4P2
Patient outcome (dead vs. alive)	LOC401127, LOC90768	LINC00937

Next, there are eight miRNAs in Table 5 having their positions in the development of CM, but none of them were related to race. Similarly, as the lncRNAs mentioned above, some miRNAs (hsa‐miR‐194‐5p, hsa‐miR‐194‐3p, hsa‐miR‐193b‐3p, and hsa‐miR‐3677‐3p) were proved to have associations with more than one clinical feature.

**Table 5:** The correlation between specific miRNAs from the ceRNA network and clinical features of CM patients

Clinical features	Upregulated miRNAs	Downregulated miRNAs
Gender (Female vs. Male)	hsa‐miR‐194‐5p	
Race (White vs. Asian)		
Tumor pathological stage (III & IV vs. I & II)	hsa‐miR‐133a‐3p	
TNM staging system (T3 + T4 vs. T1 + T2)	hsa‐miR‐194‐3p, hsa‐miR‐193b‐3p	hsa‐miR‐3677‐3p
Tumor metastasis (metastatic tumor vs. primary solid tumor)	hsa‐miR‐3917, hsa‐miR‐194‐5p	hsa‐miR‐194‐3p, hsa‐miR‐193b‐3p
Patient outcome (dead vs. alive)	hsa‐miR‐3677‐3p	hsa‐miR‐194‐3p, hsa‐miR‐708‐5p

Last, each clinical feature with its related mRNAs and the relationships are shown in Table 6, including 37 mRNAs in total. Among them, 10 mRNAs (APOE, ZNF808, TMEM65, EEF2, ELMSAN1, CLN8, 8MP8A, DIP2C, MSR1, and GNB4) were associated with two clinical features, and four mRNAs (IL21R, FCGR3A, CXCL11, and CCR5) had connections with three clinical features, not to mention the star gene, ADAMDEC1, which had an association with the tumor pathological stage, TNM staging system, tumor metastasis, and patients' outcome.

**Table 6:** The correlation between specific mRNAs from the ceRNA network and clinical features of CM patients

Clinical features	Upregulated mRNAs	Downregulated mRNAs
Gender (Female vs. Male)	PRKX, APOE	ZNF808, TMEM65, ZMYND11
Race (White vs. Asian)	CCDC25, LEPROTL1	TMEM65
Tumor pathological stage (III & IV vs. I & II)	ADAMDEC1, IL21R	EEF2, ELMSAN1
TNM staging system (T3 + T4 vs. T1 + T2)	CLN8, BMP8A, DIP2C	FCGR3A, ADAMDEC1, MSR1, CXCL11, IL21R, EEF2, CCR5, GNB4, LGSN
Tumor metastasis (metastatic tumor vs. primary solid tumor)	FCGR3A, IL21R, ADAMDEC1, MSR1, CCR5, AMIGO2, GNB4, CXCL11, CEMIP, ZNF385B	KCNK10, NME6, CLN8, TBC1D7, DIP2C, CHCHD7, PAG1, ZNF808, ELMSAN1, BMP8A, POU3F3
Patients' outcome (dead vs. alive)	VGLL3, DIEXF, ZNF471, REEP1, ZNF813, TBX3, ZNF234, ZNF28	CXCL11, IL21R, CCR5, ADAMDEC1, APOE, FCGR3A

**Figure 7:**
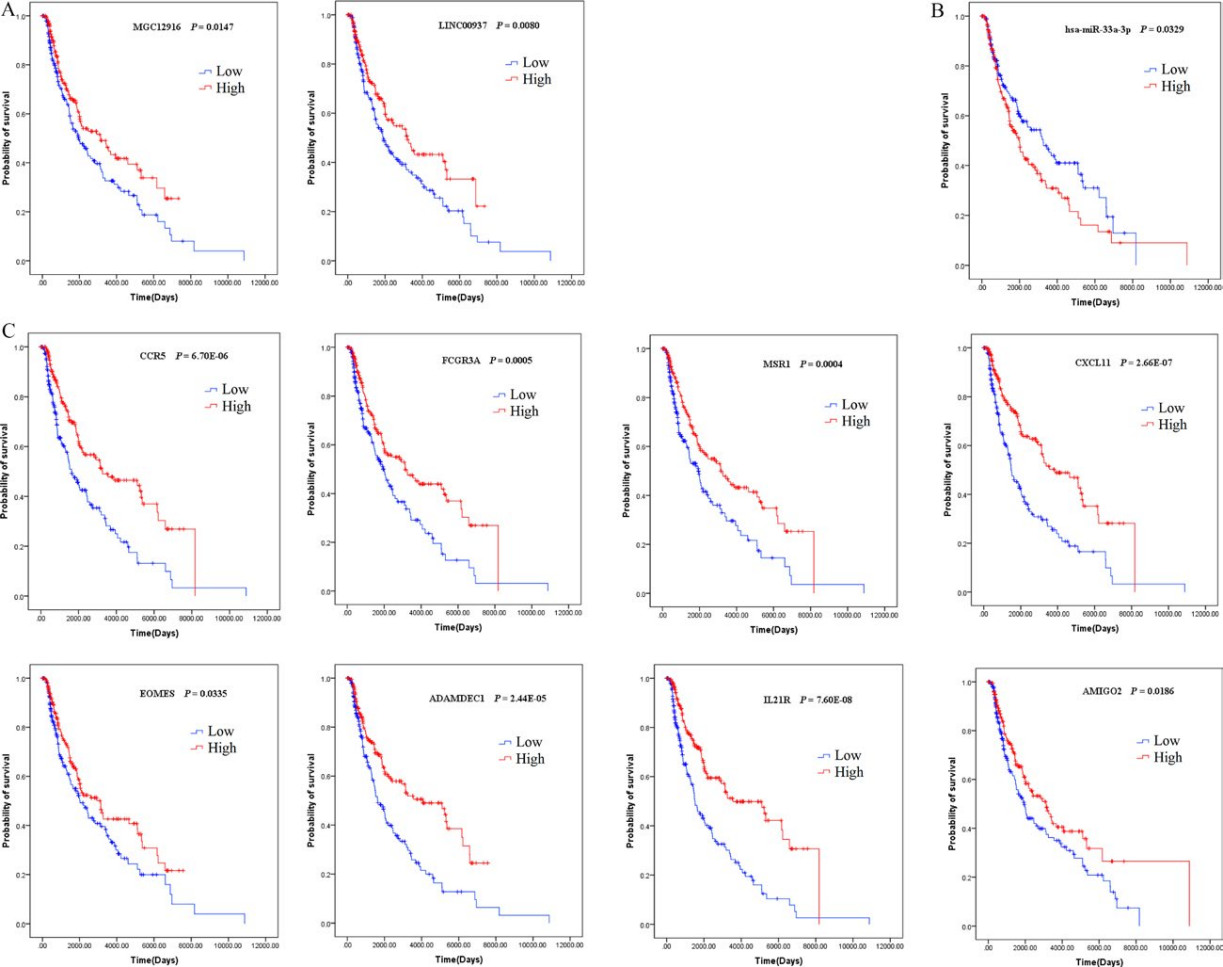
Kaplan–Meier survival curves for two lncRNAs (A), one miRNA (B), and six mRNAs (C) associated with overall survival.

To illustrate the unambiguous details about the relationship between these key genes in the ceRNA network and the outcome of CM patients, the univariate Cox proportional hazards regression model was performed, which elaborated the information of overall survival. The results implicated the expression of two lncRNAs, one miRNA and eight mRNAs, which were statistically different, as shown in Figure 7. Apparently, except for the only key miRNA, the more the other 10 genes were expressed, the longer the patients' survival.

## Discussion

To uncover the expression patterns of lncRNAs in the carcinomatosis of CM and explore the possibility of lncRNAs as biomarkers that somewhat assist the clinical practice, the TCGA database was employed. The bioinformatics analysis of RNA sequencing data of CM from TCGA provided the ceRNA network with descriptions of the interplay between key lncRNAs and other genes. In addition, when these genes were put into enrichment analysis, the results implied that immunization was considered to play a crucial role in the development of CM, as plenty of pathways and functions were associated with immunity. To date, dozens of pathways, such as the integrin/FAK signaling pathway 21, the KMT2A/hTERT signaling pathway 22, the PI3K/AKT/mTOR autophagy signaling pathway 23, and the Wnt signaling pathway 24, have been determined to somewhat govern the development of CM by disrupting the biological functions of melanocytes. Further statistical analysis of the association between gene expression and the clinical information of CM highlighted several genes, including two lncRNAs, which may influence the outcome of CM patients.

To date, there are a number of studies about microRNAs and CM 25, 26, 27, 28 but fewer about lncRNAs and CM. LncRNAs ANRIL 29, BANCR 30, GAS5 31, 32, MALAT1 33, PVT1 34, 35, SAMMON 36, 37, SPRIGHTLY 38, 39, SPRY4‐1T1 40, 41, and other lncRNAs have been studied for the regulation of melanocyte proliferation, migration, invasion, and other cell biological functions associated with the development and metastasis of CM. However, there are few studies that have finally drawn an integrative and systematic interaction of genes. Zhang et al. 42 downloaded information about CM patients and RNA sequencing data from TCGA. Although the routines and methods of bioinformatics and survival analysis are similar with this study, the results of their study determined two regulatory networks of prognostic risk lncRNAs and microRNAs, their target genes, and a PPI network. Because of the addition of the E‐MTAB‐1862 dataset and the different division of groups, there seemed to be no resemblance between the results of their study and this one. Although they have conducted quantitative real‐time polymerase chain reaction to validate the results of their bioinformatics analysis, the results of the experiments should be more convincing with more samples, which will be the next step of this study.

The survival analysis spotted two lncRNAs: MGC12916 and LINC00397. Unfortunately, MGC12916 had nothing to do with other clinical information, and LINC00397 had an association with the outcome of CM patients, which is consistent with the survival analysis. In addition, these two lncRNAs are protective factors of metastasis in CM. No studies on these two lncRNAs have been published.

However, with the inclusion of lncRNAs in the ceRNA network, there were three mRNAs that were statistically significant in the survival analysis and directly linked with hsa‐miR‐33a‐3p (CCR5, CXCL11, and ADAMDEC1) and hsa‐miR‐106b‐5p (AMIGO2, IL21R, and MSR1), of which hsa‐miR‐33a‐3p was the risk factor from the survival analysis. This may hint that perhaps the dysregulation of mRNAs has more consequential effects on the development of CM. Still, the ceRNA network provided an outstanding map to explore key genes, which can serve as biomarkers for the prognosis and carcinomatosis of CM.

## Supporting information


**Table S1.** Predictions of miRNA‐lncRNA base pairing in miRanda tools.

**Table S2.** Predictions of miRNA–mRNA base pairing in miRanda tools.
