# Supplementary material for: Integrative analysis of competing endogenous RNA network focusing on long noncoding RNA associated with progression of cutaneous melanoma
Source: Cancer Med. 2018 Mar 9;7(4):1019–29. doi: 10.1002/cam4.1315 (PMC5911588; doi:10.1002/cam4.1315)
Supplement: Supplementary file 1 — Table S1. Predictions of miRNA‐lncRNA base pairing in miRanda tools. [file CAM4-7-1019-s001.doc]

| **Supplement table1.** Predictions of miRNA-lncRNA base pairing in miRanda tools | | | | | | | | | | | |
| --- | --- | --- | --- | --- | --- | --- | --- | --- | --- | --- | --- |
| miRNA | transcrip tomelocation | lncRNA | align score | energy | miRNA start | miRNA end | gene start | gene end | miRNA_align | alignment | lncRNA_align |
| hsa-miR-106b-5p | NR_047532 | CDKN2B-AS1 | 140 | -15.32 | 3 | 17 | 1512 | 1532 | 3 uagacGUGACAGUCGUGAAau 5 | ::||| :||||||| | 5 aacatTGCTGCTAGCACTTcc 3 |
|  | NR_047534 |  | 140 | -15.32 | 3 | 17 | 776 | 796 | 3 uagacGUGACAGUCGUGAAau 5 | ::\|\|\| :\|\|\|\|\|\|\| | 5 aacatTGCTGCTAGCACTTcc 3 |
|  | NR_047535 |  | 140 | -15.32 | 3 | 17 | 805 | 825 | 3 uagacGUGACAGUCGUGAAau 5 | ::\|\|\| :\|\|\|\|\|\|\| | 5 aacatTGCTGCTAGCACTTcc 3 |
|  | NR_047536 |  | 140 | -15.32 | 3 | 17 | 669 | 689 | 3 uagacGUGACAGUCGUGAAau 5 | ::\|\|\| :\|\|\|\|\|\|\| | 5 aacatTGCTGCTAGCACTTcc 3 |
|  | NR_047543 |  | 140 | -15.32 | 3 | 17 | 805 | 825 | 3 uagacGUGACAGUCGUGAAau 5 | ::\|\|\| :\|\|\|\|\|\|\| | 5 aacatTGCTGCTAGCACTTcc 3 |
|  | NR_003529 |  | 140 | -15.32 | 3 | 17 | 2723 | 2743 | 3 uagacGUGACAGUCGUGAAau 5 | ::\|\|\| :\|\|\|\|\|\|\| | 5 aacatTGCTGCTAGCACTTcc 3 |
|  | NR_026790 | HCG11 | 159 | -16.49 | 2 | 20 | 3673 | 3693 | 3 uaGACGUGACAGUCGUGAAAu 5 | \|\|\|:\| \| ::\|\|\|\|\|\|\| | 5 tcCTGTAATCCTGGCACTTTg 3 |
|  | NR_024278 | LOC646762 | 140 | -16.81 | 3 | 19 | 3259 | 3281 | 3 uagACGU-GACAG-UCGUGAAau 5 | \|\|\|\| :\|\| \| \|\|\|\|\|\|\| | 5 acaTGCATTTGGCAAGCACTTat 3 |
|  | NR_003572 | RPL23AP53 | 151 | -17.2 | 2 | 20 | 5279 | 5299 | 3 uaGACGUGACAGUCGUGAAAu 5 | :\| \| :\|\|\|:\|\|\|\|:\|\|\| | 5 acTTCCTTTGTTAGCATTTTt 3 |
|  | NR_033708 | TP73-AS1 | 154 | -20.11 | 2 | 20 | 108 | 129 | 3 uaGAC-GUGACAGUCGUGAAAu 5 | \|\|\| \|: \| \|:\|\|\|\|\|\|\| | 5 ggCTGACGGCGGCGGCACTTTg 3 |
|  | NR_033708 |  | 147 | -16.45 | 2 | 16 | 1755 | 1775 | 3 uagacgUGACAGUCGUGAAAu 5 | \|\| \|:\|\|\|\|\|\|\| | 5 tcaacgACCAGCGGCACTTTg 3 |
|  | NR_033709 |  | 154 | -20.11 | 2 | 20 | 108 | 129 | 3 uaGAC-GUGACAGUCGUGAAAu 5 | \|\|\| \|: \| \|:\|\|\|\|\|\|\| | 5 ggCTGACGGCGGCGGCACTTTg 3 |
|  | NR_033709 |  | 147 | -16.45 | 2 | 16 | 1755 | 1775 | 3 uagacgUGACAGUCGUGAAAu 5 | \|\| \|:\|\|\|\|\|\|\| | 5 tcaacgACCAGCGGCACTTTg 3 |
|  | NR_033710 |  | 154 | -20.11 | 2 | 20 | 108 | 129 | 3 uaGAC-GUGACAGUCGUGAAAu 5 | \|\|\| \|: \| \|:\|\|\|\|\|\|\| | 5 ggCTGACGGCGGCGGCACTTTg 3 |
|  | NR_033710 |  | 147 | -16.45 | 2 | 16 | 1755 | 1775 | 3 uagacgUGACAGUCGUGAAAu 5 | \|\| \|:\|\|\|\|\|\|\| | 5 tcaacgACCAGCGGCACTTTg 3 |
|  | NR_033711 |  | 154 | -20.11 | 2 | 20 | 108 | 129 | 3 uaGAC-GUGACAGUCGUGAAAu 5 | \|\|\| \|: \| \|:\|\|\|\|\|\|\| | 5 ggCTGACGGCGGCGGCACTTTg 3 |
|  | NR_033711 |  | 147 | -16.45 | 2 | 16 | 1755 | 1775 | 3 uagacgUGACAGUCGUGAAAu 5 | \|\| \|:\|\|\|\|\|\|\| | 5 tcaacgACCAGCGGCACTTTg 3 |
|  | NR_033712 |  | 147 | -16.45 | 2 | 16 | 1048 | 1068 | 3 uagacgUGACAGUCGUGAAAu 5 | \|\| \|:\|\|\|\|\|\|\| | 5 tcaacgACCAGCGGCACTTTg 3 |
|  | NR_037805 | ZNF321P | 151 | -18.55 | 2 | 20 | 2072 | 2092 | 3 uaGACGUGACAGUCGUGAAAu 5 | \|\|\|:\| \| \|\|\|\|\|:\|\|\| | 5 gcCTGTAATCCCAGCATTTTg 3 |
| hsa-miR-133a-3p | NR_015399 | LINC01102 | 145 | -31.62 | 2 | 21 | 663 | 685 | 3 guCGACCAACUUCC--CCUGGUUu 5 | ||||| ||:|| ||||||: | 5 caGCTGG-CGAGGGGAGGACCAGg 3 |
|  | NR_037805 | ZNF321P | 148 | -18.8 | 2 | 17 | 2265 | 2286 | 3 gucgacCAACUUCCCCUGGUUu 5 | \|\|\|\| \|\| \|\|:\|\|\|\| | 5 gtggaaGTTGCAGTGGGCCAAg 3 |
| hsa-miR-193b-3p | NR_024125 | ATP1A1-AS1 | 145 | -18.2 | 2 | 10 | 536 | 557 | 3 ucgcccugaaacuCCCGGUCAa 5 | |||||||| | 5 tgccgtcacagctGGGCCAGTa 3 |
|  | NR_024126 |  | 145 | -18.2 | 2 | 10 | 299 | 320 | 3 ucgcccugaaacuCCCGGUCAa 5 | \|\|\|\|\|\|\|\| | 5 tgccgtcacagctGGGCCAGTa 3 |
|  | NR_024124 |  | 145 | -18.2 | 2 | 10 | 260 | 281 | 3 ucgcccugaaacuCCCGGUCAa 5 | \|\|\|\|\|\|\|\| | 5 tgccgtcacagctGGGCCAGTa 3 |
|  | NR_003260 | DNM1P46 | 146 | -18.9 | 2 | 16 | 2628 | 2652 | 3 ucgcccuGAAACU---CCCGGUCAa 5 | :\| \|\|\| \|\|\|\|\|\|\|\| | 5 gctccctTTCTGACCTGGGCCAGTt 3 |
|  | NR_027000 | LINC00965 | 149 | -20.17 | 2 | 20 | 1588 | 1608 | 3 ucgCCCUGAAACUCCCGGUCAa 5 | \|\|\| \|\| \| \|\|\|\|\|\|\| | 5 ctgGGGTCTAT-TTGGCCAGTg 3 |
|  | NR_027000 |  | 141 | -21.85 | 2 | 20 | 2793 | 2813 | 3 ucgCCCUGAAACUCCCGGUCAa 5 | \|\| \|\|\| \|\| \|\|\|\|:\|\| | 5 gagGGAACTCAGA-GGCCGGTg 3 |
|  | NR_026922 | LOC150776 | 140 | -20.38 | 3 | 17 | 1878 | 1899 | 3 ucgcccUGAAACUCCCGGUCaa 5 | :\| \| \|\|\|\|\|\|\|\|\| | 5 tctacaGCCTGGAGGGCCAGga 3 |
|  | NR_026854 | LOC401127 | 140 | -18.85 | 2 | 9 | 803 | 824 | 3 ucgcccugaaacucCCGGUCAa 5 | \|\|\|\|\|\|\| | 5 gggacacagcctcaGGCCAGTg 3 |
|  | NR_028092 | LPAL2 | 156 | -21.84 | 2 | 21 | 91 | 112 | 3 ucGCCCUGAAACUCCCGGUCAa 5 | \| \|\|:\| \|\| \|\|\|\|\|\|\| | 5 ttCTGGGCACTGCTGGCCAGTc 3 |
|  | NR_028093 |  | 156 | -21.84 | 2 | 21 | 91 | 112 | 3 ucGCCCUGAAACUCCCGGUCAa 5 | \| \|\|:\| \|\| \|\|\|\|\|\|\| | 5 ttCTGGGCACTGCTGGCCAGTc 3 |
|  | NR_026880 | MGC12916 | 140 | -24.13 | 3 | 21 | 28 | 49 | 3 ucGCCCUGAAACUCCCGGUCaa 5 | :\|\| : \|\| \|\|\|\|\|\|\|\| | 5 gaTGGTGATTCCAGGGCCAGgc 3 |
|  | NR_027275 | SRRM2-AS1 | 140 | -17.05 | 2 | 9 | 115 | 136 | 3 ucgcccugaaacucCCGGUCAa 5 | \|\|\|\|\|\|\| | 5 ctaagcgtcccgttGGCCAGTg 3 |
|  | NR_038327 | TEKT4P2 | 146 | -19.31 | 2 | 13 | 1079 | 1099 | 3 ucgcccugaaACUCCCGGUCAa 5 | \|\|\| \|\|\|\|\|\|\| | 5 cctggtcggcTGA-GGCCAGTc 3 |
|  | NR_104604 | TTLL13P | 144 | -28.49 | 3 | 20 | 193 | 214 | 3 ucgCCCUGAAACUC-CCGGUCaa 5 | \|\|\|\|\| \|\|\|\| \|\|\|\|\|\| | 5 gagGGGAC-ATGAGCGGCCAGct 3 |
| hsa-miR-194-3p | NR_047539 | CDKN2B-AS1 | 141 | -18.33 | 2 | 20 | 2367 | 2387 | 3 gucUAUUGUCGUCGGGGUGACc 5 | ||:|: ||| |:||||| | 5 ggcATGATTACAG-CTCACTGc 3 |
|  | NR_047540 |  | 141 | -18.33 | 2 | 20 | 967 | 987 | 3 gucUAUUGUCGUCGGGGUGACc 5 | \|\|:\|: \|\|\| \|:\|\|\|\|\| | 5 ggcATGATTACAG-CTCACTGc 3 |
|  | NR_003529 |  | 141 | -18.33 | 2 | 20 | 2367 | 2387 | 3 gucUAUUGUCGUCGGGGUGACc 5 | \|\|:\|: \|\|\| \|:\|\|\|\|\| | 5 ggcATGATTACAG-CTCACTGc 3 |
|  | NR_026790 | HCG11 | 147 | -21.69 | 2 | 17 | 154 | 177 | 3 gucuauUGUCGUCG--GGGUGACc 5 | :\| \|::\|\| \|\|\|\|\|\|\| | 5 gcctctGCTGTGGCTGCCCACTGg 3 |
|  | NR_026790 |  | 140 | -19.88 | 2 | 9 | 1599 | 1620 | 3 gucuauugucgucgGGGUGACc 5 | \|\|\|\|\|\|\| | 5 tttgagcattcttgCCCACTGg 3 |
|  | NR_024278 | LOC646762 | 140 | -28.67 | 4 | 21 | 2202 | 2223 | 3 guCUAUUGUCGUCGGGGUGacc 5 | \|:\|:\| :\|\|\|\|\|\|\|\|\|\| | 5 ttGGTGAGGGCAGCCCCACgca 3 |
|  | NR_033708 | TP73-AS1 | 154 | -18.44 | 2 | 20 | 3887 | 3909 | 3 gucUAUUG-UCGUCGGGGUGACc 5 | \| \|\|\| \| \| \|\|\|\|\|\|\|\| | 5 acaAAAACAAAAATCCCCACTGa 3 |
|  | NR_033708 |  | 147 | -22.31 | 2 | 21 | 1094 | 1111 | 3 guCUAUUGUCGUCGGGGUGACc 5 | \|:\|\|\|\| \|\|\|\|\|\|\|\| | 5 agGGTAAC----TCCCCACTGt 3 |
|  | NR_033708 |  | 140 | -19.57 | 2 | 21 | 1205 | 1226 | 3 guCUAUUGUCGUCGGGGUGACc 5 | \| \|: \|\| \|\|\|\|\|::\|\|\|\| | 5 tcGCTGCCACCAGCCTTACTGc 3 |
|  | NR_033709 |  | 154 | -18.44 | 2 | 20 | 4500 | 4522 | 3 gucUAUUG-UCGUCGGGGUGACc 5 | \| \|\|\| \| \| \|\|\|\|\|\|\|\| | 5 acaAAAACAAAAATCCCCACTGa 3 |
|  | NR_033709 |  | 147 | -22.31 | 2 | 21 | 1094 | 1111 | 3 guCUAUUGUCGUCGGGGUGACc 5 | \|:\|\|\|\| \|\|\|\|\|\|\|\| | 5 agGGTAAC----TCCCCACTGt 3 |
|  | NR_033709 |  | 140 | -19.57 | 2 | 21 | 1205 | 1226 | 3 guCUAUUGUCGUCGGGGUGACc 5 | \| \|: \|\| \|\|\|\|\|::\|\|\|\| | 5 tcGCTGCCACCAGCCTTACTGc 3 |
|  | NR_033710 |  | 154 | -18.44 | 2 | 20 | 5755 | 5777 | 3 gucUAUUG-UCGUCGGGGUGACc 5 | \| \|\|\| \| \| \|\|\|\|\|\|\|\| | 5 acaAAAACAAAAATCCCCACTGa 3 |
|  | NR_033710 |  | 147 | -22.31 | 2 | 21 | 1094 | 1111 | 3 guCUAUUGUCGUCGGGGUGACc 5 | \|:\|\|\|\| \|\|\|\|\|\|\|\| | 5 agGGTAAC----TCCCCACTGt 3 |
|  | NR_033710 |  | 140 | -19.57 | 2 | 21 | 1205 | 1226 | 3 guCUAUUGUCGUCGGGGUGACc 5 | \| \|: \|\| \|\|\|\|\|::\|\|\|\| | 5 tcGCTGCCACCAGCCTTACTGc 3 |
|  | NR_033711 |  | 154 | -18.44 | 2 | 20 | 6219 | 6241 | 3 gucUAUUG-UCGUCGGGGUGACc 5 | \| \|\|\| \| \| \|\|\|\|\|\|\|\| | 5 acaAAAACAAAAATCCCCACTGa 3 |
|  | NR_033711 |  | 147 | -22.31 | 2 | 21 | 1094 | 1111 | 3 guCUAUUGUCGUCGGGGUGACc 5 | \|:\|\|\|\| \|\|\|\|\|\|\|\| | 5 agGGTAAC----TCCCCACTGt 3 |
|  | NR_033711 |  | 140 | -19.57 | 2 | 21 | 1205 | 1226 | 3 guCUAUUGUCGUCGGGGUGACc 5 | \| \|: \|\| \|\|\|\|\|::\|\|\|\| | 5 tcGCTGCCACCAGCCTTACTGc 3 |
|  | NR_033712 |  | 154 | -18.44 | 2 | 20 | 3134 | 3156 | 3 gucUAUUG-UCGUCGGGGUGACc 5 | \| \|\|\| \| \| \|\|\|\|\|\|\|\| | 5 acaAAAACAAAAATCCCCACTGa 3 |
|  | NR_033712 |  | 140 | -19.57 | 2 | 21 | 498 | 519 | 3 guCUAUUGUCGUCGGGGUGACc 5 | \| \|: \|\| \|\|\|\|\|::\|\|\|\| | 5 tcGCTGCCACCAGCCTTACTGc 3 |
| hsa-miR-194-5p | NR_003572 | RPL23AP53 | 143 | -17.94 | 2 | 19 | 1739 | 1758 | 3 agguGUACCUCAACGACAAUGu 5 | |||| || |:|||||| | 5 gaatCATG--GTGGTTGTTACt 3 |
|  | NR_033709 | TP73-AS1 | 152 | -14.79 | 2 | 13 | 2356 | 2377 | 3 agguguaccuCAACGACAAUGu 5 | \|\|\| \|\|\|\|\|\|\| | 5 tgtttcacttGTTACTGTTACt 3 |
|  | NR_033710 |  | 152 | -14.79 | 2 | 13 | 3611 | 3632 | 3 agguguaccuCAACGACAAUGu 5 | \|\|\| \|\|\|\|\|\|\| | 5 tgtttcacttGTTACTGTTACt 3 |
|  | NR_033711 |  | 152 | -14.79 | 2 | 13 | 4075 | 4096 | 3 agguguaccuCAACGACAAUGu 5 | \|\|\| \|\|\|\|\|\|\| | 5 tgtttcacttGTTACTGTTACt 3 |
| hsa-miR-33a-3p | NR_047532 | CDKN2B-AS1 | 145 | -16.08 | 2 | 10 | 1497 | 1518 | 3 cacuacgugacacCUUUGUAAc 5 | |||||||| | 5 tctcacatggcaaGAAACATTg 3 |
|  | NR_047534 |  | 145 | -16.08 | 2 | 10 | 761 | 782 | 3 cacuacgugacacCUUUGUAAc 5 | \|\|\|\|\|\|\|\| | 5 tctcacatggcaaGAAACATTg 3 |
|  | NR_047535 |  | 145 | -16.08 | 2 | 10 | 790 | 811 | 3 cacuacgugacacCUUUGUAAc 5 | \|\|\|\|\|\|\|\| | 5 tctcacatggcaaGAAACATTg 3 |
|  | NR_047536 |  | 145 | -16.08 | 2 | 10 | 654 | 675 | 3 cacuacgugacacCUUUGUAAc 5 | \|\|\|\|\|\|\|\| | 5 tctcacatggcaaGAAACATTg 3 |
|  | NR_047543 |  | 145 | -16.08 | 2 | 10 | 790 | 811 | 3 cacuacgugacacCUUUGUAAc 5 | \|\|\|\|\|\|\|\| | 5 tctcacatggcaaGAAACATTg 3 |
|  | NR_003529 |  | 145 | -16.08 | 2 | 10 | 2708 | 2729 | 3 cacuacgugacacCUUUGUAAc 5 | \|\|\|\|\|\|\|\| | 5 tctcacatggcaaGAAACATTg 3 |
| hsa-miR-3677-3p | NR_047539 | CDKN2B-AS1 | 140 | -19.95 | 2 | 14 | 2099 | 2121 | 3 ccggcaccgGUC-UCGGGUGCUc 5 | ||| ||||:|||| | 5 taatttaaaCAGAAGCCTACGAa 3 |
|  | NR_047540 |  | 140 | -19.95 | 2 | 14 | 795 | 817 | 3 ccggcaccgGUC-UCGGGUGCUc 5 | \|\|\| \|\|\|\|:\|\|\|\| | 5 taatttaaaCAGAAGCCTACGAa 3 |
|  | NR_003529 |  | 140 | -19.95 | 2 | 14 | 2099 | 2121 | 3 ccggcaccgGUC-UCGGGUGCUc 5 | \|\|\| \|\|\|\|:\|\|\|\| | 5 taatttaaaCAGAAGCCTACGAa 3 |
|  | NR_015399 | LINC01102 | 144 | -17.86 | 2 | 13 | 329 | 350 | 3 ccggcaccggUCUCGGGUGCUc 5 | \|\| \|\|\|\|\|\|\| | 5 tcatgagtgaAGTACCCACGAt 3 |
|  | NR_033708 | TP73-AS1 | 150 | -36.61 | 2 | 21 | 558 | 581 | 3 ccGGCAC-CG-GUCUCGGGUGCUc 5 | \| \|\|\| \|\| \|\|\|\| \|\|\|\|\|\|: | 5 ggCGGTGAGCGCAGATCCCACGGg 3 |
|  | NR_033709 |  | 150 | -36.61 | 2 | 21 | 558 | 581 | 3 ccGGCAC-CG-GUCUCGGGUGCUc 5 | \| \|\|\| \|\| \|\|\|\| \|\|\|\|\|\|: | 5 ggCGGTGAGCGCAGATCCCACGGg 3 |
|  | NR_033710 |  | 150 | -36.61 | 2 | 21 | 558 | 581 | 3 ccGGCAC-CG-GUCUCGGGUGCUc 5 | \| \|\|\| \|\| \|\|\|\| \|\|\|\|\|\|: | 5 ggCGGTGAGCGCAGATCCCACGGg 3 |
|  | NR_033711 |  | 150 | -36.61 | 2 | 21 | 558 | 581 | 3 ccGGCAC-CG-GUCUCGGGUGCUc 5 | \| \|\|\| \|\| \|\|\|\| \|\|\|\|\|\|: | 5 ggCGGTGAGCGCAGATCCCACGGg 3 |
| hsa-miR-3917 | NR_024420 | LINC00937 | 154 | -27.41 | 2 | 19 | 13 | 32 | 3 ggGUGGACGAGUCAGGCUCg 5 | |: |||||::|||||:| | 5 agCGACTGCTTGGTCCGGGg 3 |
|  | NR_033708 | TP73-AS1 | 142 | -27.51 | 2 | 17 | 3807 | 3825 | 3 ggguGGACGAGUCAGGCUCg 5 | \|\|\|\|:\|: \|\|\|:\|\|\| | 5 ccgcCCTGTTT-GTCTGAGc 3 |
|  | NR_033709 |  | 142 | -27.51 | 2 | 17 | 4420 | 4438 | 3 ggguGGACGAGUCAGGCUCg 5 | \|\|\|\|:\|: \|\|\|:\|\|\| | 5 ccgcCCTGTTT-GTCTGAGc 3 |
|  | NR_033710 |  | 142 | -27.51 | 2 | 17 | 5675 | 5693 | 3 ggguGGACGAGUCAGGCUCg 5 | \|\|\|\|:\|: \|\|\|:\|\|\| | 5 ccgcCCTGTTT-GTCTGAGc 3 |
|  | NR_033711 |  | 142 | -27.51 | 2 | 17 | 6139 | 6157 | 3 ggguGGACGAGUCAGGCUCg 5 | \|\|\|\|:\|: \|\|\|:\|\|\| | 5 ccgcCCTGTTT-GTCTGAGc 3 |
|  | NR_033712 |  | 142 | -27.51 | 2 | 17 | 3054 | 3072 | 3 ggguGGACGAGUCAGGCUCg 5 | \|\|\|\|:\|: \|\|\|:\|\|\| | 5 ccgcCCTGTTT-GTCTGAGc 3 |
| hsa-miR-658 | NR_047532 | CDKN2B-AS1 | 144 | -25.89 | 2 | 23 | 1258 | 1281 | 3 uggUUGCCUGGAUGAAGGGAGGCGg 5 | ||| ||| |:| ||||||| | | 5 agaAACAGACATGC-TCCCTCCCCt 3 |
|  | NR_003529 |  | 144 | -25.89 | 2 | 23 | 2469 | 2492 | 3 uggUUGCCUGGAUGAAGGGAGGCGg 5 | \|\|\| \|\|\| \|:\| \|\|\|\|\|\|\| \| | 5 agaAACAGACATGC-TCCCTCCCCt 3 |
|  | NR_024278 | LOC646762 | 155 | -32.29 | 2 | 12 | 844 | 868 | 3 ugguugccuggaugAAGGGAGGCGg 5 | \|\|\|\|\|\|\|\|\|\| | 5 caccccctggcaggTTCCCTCCGCc 3 |
|  | NR_024278 |  | 155 | -32.29 | 2 | 12 | 1657 | 1681 | 3 ugguugccuggaugAAGGGAGGCGg 5 | \|\|\|\|\|\|\|\|\|\| | 5 cgccccctggcaggTTCCCTCCGCc 3 |
|  | NR_024278 |  | 152 | -24.82 | 2 | 23 | 1892 | 1915 | 3 uggUUGCCUGGAUGAAGGGAGGCGg 5 | \|\|:\|\| :\|\| : \|\|\|\|\|\|\| | 5 tgaAATGGCTCT-TGAGCCTCCGCt 3 |
|  | NR_024278 |  | 146 | -22.87 | 2 | 21 | 1633 | 1656 | 3 ugguuGCCUGGAUGAAGGGAGGCGg 5 | \| \|\| :\|:\| \|\|\|\|\|\|\| | 5 ggtctCAGA-TTGCGGACCTCCGCa 3 |
|  | NR_024278 |  | 144 | -20.39 | 2 | 24 | 1552 | 1574 | 3 ugGUUGCCUGGAUGAAGGGAGGCGg 5 | \|\| \| \|\|\| \| \|\|\|\|\|\|\| | 5 cgCACAGCACC--CCAGCCTCCGCg 3 |
|  | NR_024278 |  | 142 | -22.72 | 2 | 23 | 5009 | 5031 | 3 uggUUGCCUGGAUGAAGGGAGGCGg 5 | :\|: \|\|\| \|\|\|\| \|\|\|\|:\|\| | 5 ctaGATCCACC-ACTT-CCTCTGCt 3 |
|  | NR_033708 | TP73-AS1 | 145 | -28.92 | 3 | 24 | 351 | 374 | 3 ugGUUGCCUGGAUGAAGGGAGGCgg 5 | :\|\| \|\|\|:\| :\| \|\|\|\|\|\|\| | 5 ccTAAGGGATCCGC-CCCCTCCGag 3 |
|  | NR_033708 |  | 145 | -34.8 | 2 | 23 | 405 | 431 | 3 uggUUGCCUGGAUGAAG--GGAGGCGg 5 | :\|\|\|\|\|\|\| \|\|\| \| \|\| \|\|\|\| | 5 tcgGACGGACCCACTCCAGCCCCCGCt 3 |
|  | NR_033709 |  | 145 | -28.92 | 3 | 24 | 351 | 374 | 3 ugGUUGCCUGGAUGAAGGGAGGCgg 5 | :\|\| \|\|\|:\| :\| \|\|\|\|\|\|\| | 5 ccTAAGGGATCCGC-CCCCTCCGag 3 |
|  | NR_033709 |  | 145 | -34.8 | 2 | 23 | 405 | 431 | 3 uggUUGCCUGGAUGAAG--GGAGGCGg 5 | :\|\|\|\|\|\|\| \|\|\| \| \|\| \|\|\|\| | 5 tcgGACGGACCCACTCCAGCCCCCGCt 3 |
|  | NR_033710 |  | 145 | -28.92 | 3 | 24 | 351 | 374 | 3 ugGUUGCCUGGAUGAAGGGAGGCgg 5 | :\|\| \|\|\|:\| :\| \|\|\|\|\|\|\| | 5 ccTAAGGGATCCGC-CCCCTCCGag 3 |
|  | NR_033710 |  | 145 | -34.8 | 2 | 23 | 405 | 431 | 3 uggUUGCCUGGAUGAAG--GGAGGCGg 5 | :\|\|\|\|\|\|\| \|\|\| \| \|\| \|\|\|\| | 5 tcgGACGGACCCACTCCAGCCCCCGCt 3 |
|  | NR_033710 |  | 142 | -24.87 | 2 | 22 | 2776 | 2798 | 3 ugguUGCCUGGAUGAAGGGAGGCGg 5 | \|:\| \|\|\|: \|\|\|\|\|:\|\|\| | 5 gggcATGTCCCTG--TCCCTTCGCt 3 |
|  | NR_033711 |  | 145 | -28.92 | 3 | 24 | 351 | 374 | 3 ugGUUGCCUGGAUGAAGGGAGGCgg 5 | :\|\| \|\|\|:\| :\| \|\|\|\|\|\|\| | 5 ccTAAGGGATCCGC-CCCCTCCGag 3 |
|  | NR_033711 |  | 145 | -34.8 | 2 | 23 | 405 | 431 | 3 uggUUGCCUGGAUGAAG--GGAGGCGg 5 | :\|\|\|\|\|\|\| \|\|\| \| \|\| \|\|\|\| | 5 tcgGACGGACCCACTCCAGCCCCCGCt 3 |
|  | NR_033711 |  | 142 | -24.87 | 2 | 22 | 3240 | 3262 | 3 ugguUGCCUGGAUGAAGGGAGGCGg 5 | \|:\| \|\|\|: \|\|\|\|\|:\|\|\| | 5 gggcATGTCCCTG--TCCCTTCGCt 3 |
| hsa-miR-708-5p | NR_026756 | CYP4F35P | 149 | -16.39 | 2 | 22 | 2290 | 2314 | 3 ggGUCGAU-CUAACAU--UCGAGGAa 5 | || ||: | || || ||||||| | 5 gtCA-CTGTGTTTTTAAGAGCTCCTt 3 |
|  | NR_003260 | DNM1P46 | 145 | -17.82 | 2 | 10 | 1271 | 1293 | 3 gggucgaucuaacaUUCGAGGAa 5 | \|\|\|\|\|\|\|\| | 5 ttatcagcacccacAAGCTCCTg 3 |
|  | NR_026922 | LOC150776 | 140 | -15.6 | 3 | 21 | 654 | 676 | 3 gggUCGAUCUAACAUUCGAGGaa 5 | \|\|:\|:\| \|\| \|\|\|\|\|\|:\| | 5 tgaAGTTGGTTTATAAGCTTCaa 3 |
|  | NR_027107 | LOC90768 | 144 | -22.95 | 2 | 22 | 1074 | 1098 | 3 ggGUCGAUCUA--ACAUUCGAGGAa 5 | \|:\|\| :\|\|\| \| \|:\|\|\|\|\|\| | 5 gtCGGCGGGATCCTTCAGGCTCCTt 3 |
|  | NR_026880 | MGC12916 | 145 | -19.03 | 2 | 19 | 610 | 637 | 3 gggucGAUCUAACAU-----UCGAGGAa 5 | \|\|:\|\| \|\| \| \|\|\|\|\|\|\| | 5 cgttcCTGGACTGCACCAGCAGCTCCTg 3 |
|  | NR_038327 | TEKT4P2 | 154 | -21.45 | 2 | 22 | 806 | 826 | 3 ggGUCGAUCUAACAUUCGAGGAa 5 | \|\|\| \| \| \|\|:\|\|\|\|\|\|\| | 5 ttCAG--ACAAGGTGAGCTCCTg 3 |
|  | NR_038327 |  | 147 | -24.8 | 2 | 12 | 785 | 807 | 3 gggucgaucuaaCAUUCGAGGAa 5 | \| \|\|\|\|\|\|\|\| | 5 atcttcccgggaGGAAGCTCCTt 3 |
