# Supplementary material for: Integrative analysis of competing endogenous RNA network focusing on long noncoding RNA associated with progression of cutaneous melanoma
Source: Cancer Med. 2018 Mar 9;7(4):1019–29. doi: 10.1002/cam4.1315 (PMC5911588; doi:10.1002/cam4.1315)
Supplement: Supplementary file 2 — Table S2. Predictions of miRNA–mRNA base pairing in miRanda tools. [file CAM4-7-1019-s002.doc]

| **Supplement table2.** Predictions of miRNA-mRNA base pairing in miRanda tools | | | | | | | | | | | |
| --- | --- | --- | --- | --- | --- | --- | --- | --- | --- | --- | --- |
| miRNA | mRNA | refseq | align score | energy | miRNA start | miRNA end | gene start | gene end | miRNA_align | alignment | mRNA_align |
| hsa-miR-106b-5p | AMIGO2 | NM_181847 | 153 | -16.48 | 2 | 17 | 1075 | 1093 | 3 uagacGUGACAGUCGUGAAAu 5 | ||:||| ||||||| | 5 gatatCATTGT--GCACTTTa 3 |
|  | ARHGEF10 | NM_001308152 | 157 | -15.93 | 2 | 14 | 1209 | 1229 | 3 uagacgugACAGUCGUGAAAu 5 | \| \|\|\|\|\|\|\|\|\|\| | 5 aataaatgTCTCAGCACTTTa 3 |
|  | BNIP3L | NM_001330491 | 158 | -18.94 | 2 | 20 | 2615 | 2636 | 3 uaGACGUGA-CAGUCGUGAAAu 5 | \|\| : :\| \|\|:\|\|\|\|\|\|\|\| | 5 atCTATTTTAGTTAGCACTTTg 3 |
|  |  | NM_004331 | 158 | -18.94 | 2 | 20 | 2615 | 2636 | 3 uaGACGUGA-CAGUCGUGAAAu 5 | \|\| : :\| \|\|:\|\|\|\|\|\|\|\| | 5 atCTATTTTAGTTAGCACTTTg 3 |
|  | CCDC25 | NM_001304529 | 157 | -18.35 | 2 | 20 | 977 | 1000 | 3 uaGACGU-GACA--GUCGUGAAAu 5 | :\| \|\| \|\|\|\| :\|\|\|\|\|\|\|\| | 5 taTTCCACCTGTAATAGCACTTTg 3 |
|  |  | NM_001304530 | 157 | -18.35 | 2 | 20 | 890 | 913 | 3 uaGACGU-GACA--GUCGUGAAAu 5 | :\| \|\| \|\|\|\| :\|\|\|\|\|\|\|\| | 5 taTTCCACCTGTAATAGCACTTTg 3 |
|  |  | NM_001304532 | 157 | -18.35 | 2 | 20 | 918 | 941 | 3 uaGACGU-GACA--GUCGUGAAAu 5 | :\| \|\| \|\|\|\| :\|\|\|\|\|\|\|\| | 5 taTTCCACCTGTAATAGCACTTTg 3 |
|  | CLN8 | NM_018941 | 167 | -20.77 | 2 | 20 | 3457 | 3477 | 3 uaGACGUGACAGUCGUGAAAu 5 | \|\|\|:\| \| \|\|\|\|\|\|\|\|\| | 5 gcCTGTAATCCCAGCACTTTg 3 |
|  | CTSB | NM_001317237 | 167 | -20.77 | 2 | 20 | 1899 | 1919 | 3 uaGACGUGACAGUCGUGAAAu 5 | \|\|\|:\| \| \|\|\|\|\|\|\|\|\| | 5 gcCTGTAATCCCAGCACTTTg 3 |
|  |  | NM_001317237 | 167 | -20.77 | 2 | 20 | 2209 | 2229 | 3 uaGACGUGACAGUCGUGAAAu 5 | \|\|\|:\| \| \|\|\|\|\|\|\|\|\| | 5 gcCTGTAATCCCAGCACTTTg 3 |
|  | GNB4 | NM_021629 | 167 | -20.77 | 2 | 20 | 3205 | 3225 | 3 uaGACGUGACAGUCGUGAAAu 5 | \|\|\|:\| \| \|\|\|\|\|\|\|\|\| | 5 acCTGTAATCCCAGCACTTTg 3 |
|  | IL21R | NM_021798 | 171 | -20.69 | 2 | 20 | 2164 | 2184 | 3 uaGACGUGACAGUCGUGAAAu 5 | \|\|\|:: \| \|\|\|\|\|\|\|\|\|\| | 5 acCTGTGATCTCAGCACTTTg 3 |
|  | MSR1 | NM_138715 | 153 | -16.04 | 2 | 15 | 714 | 735 | 3 uagacguGACAGU-CGUGAAAu 5 | :\|\|\|:\| \|\|\|\|\|\|\| | 5 gtttagtTTGTTATGCACTTTa 3 |
|  | NME6 | NM_001308426 | 159 | -15.35 | 2 | 20 | 286 | 306 | 3 uaGACGUGACAGUCGUGAAAu 5 | \|\| :\| \| \|\|\|\|\|\|\|\|\| | 5 gcCTATAATCCCAGCACTTTg 3 |
|  |  | NM_001308430 | 159 | -15.35 | 2 | 20 | 315 | 335 | 3 uaGACGUGACAGUCGUGAAAu 5 | \|\| :\| \| \|\|\|\|\|\|\|\|\| | 5 gcCTATAATCCCAGCACTTTg 3 |
|  | PAG1 | NM_018440 | 167 | -20.77 | 2 | 20 | 6153 | 6173 | 3 uaGACGUGACAGUCGUGAAAu 5 | \|\|\|:\| \| \|\|\|\|\|\|\|\|\| | 5 gcCTGTAATCCCAGCACTTTg 3 |
|  | PRKX | NM_005044 | 167 | -20.77 | 2 | 20 | 2315 | 2335 | 3 uaGACGUGACAGUCGUGAAAu 5 | \|\|\|:\| \| \|\|\|\|\|\|\|\|\| | 5 gcCTGTAATCCCAGCACTTTg 3 |
|  |  | NM_005044 | 159 | -16.01 | 2 | 20 | 808 | 828 | 3 uaGACGUGACAGUCGUGAAAu 5 | \|\| :\| \| \|\|\|\|\|\|\|\|\| | 5 tcCTATAGTCCCAGCACTTTg 3 |
|  | RAB11FIP1 | NM_001002814 | 167 | -20.77 | 2 | 20 | 1027 | 1047 | 3 uaGACGUGACAGUCGUGAAAu 5 | \|\|\|:\| \| \|\|\|\|\|\|\|\|\| | 5 gcCTGTAATCCCAGCACTTTg 3 |
|  |  | NM_001002814 | 167 | -20.77 | 2 | 20 | 2269 | 2289 | 3 uaGACGUGACAGUCGUGAAAu 5 | \|\|\|:\| \| \|\|\|\|\|\|\|\|\| | 5 gcCTGTAATCCCAGCACTTTg 3 |
|  | RAB31 | NM_006868 | 169 | -21.59 | 2 | 18 | 986 | 1006 | 3 uagaCGUGACAGUCGUGAAAu 5 | \| \| \|\|\|\|\|\|\|\|\|\|\|\| | 5 agagGAAGTGTCAGCACTTTa 3 |
|  | TBX3 | NM_005996 | 150 | -16.72 | 2 | 16 | 35 | 56 | 3 uagacgUGACAGU-CGUGAAAu 5 | :\|\|\| \|: \|\|\|\|\|\|\| | 5 gttcagGCTGCCGTGCACTTTg 3 |
|  | TNFRSF21 | NM_014452 | 154 | -17.4 | 2 | 17 | 840 | 859 | 3 uagacGUGACAGUCGUGAAAu 5 | \|\|:\|\| \| \|\|\|\|\|\|\| | 5 gaacaCATTG-CTGCACTTTg 3 |
|  | ZNF234 | NM_001144824 | 159 | -20.87 | 2 | 20 | 1653 | 1673 | 3 uaGACGUGACAGUCGUGAAAu 5 | \|\|\|:\| \|\|\|\|\|\|\|\|\| | 5 gcCTGTAACCCCAGCACTTTa 3 |
|  | ZNF28 | NM_006969 | 179 | -24.71 | 2 | 20 | 1409 | 1429 | 3 uaGACGUGACAGUCGUGAAAu 5 | \|\|\|\|\| \|\| \|\|\|\|\|\|\|\|\| | 5 gcCTGCAATGCCAGCACTTTg 3 |
|  | ZNF471 | NM_001321768 | 154 | -22.79 | 2 | 15 | 3934 | 3954 | 3 uagacguGACAGUCGUGAAAu 5 | \|\|\|\|\|\|\|\|\|:\|\|\| | 5 gtgttacCTGTCAGCATTTTg 3 |
|  | ZNF813 | NM_001004301 | 167 | -20.77 | 2 | 20 | 1043 | 1063 | 3 uaGACGUGACAGUCGUGAAAu 5 | \|\|\|:\| \| \|\|\|\|\|\|\|\|\| | 5 acCTGTAATCCCAGCACTTTg 3 |
| hsa-miR-133a-3p | CXCL11 | NM_001302123 | 162 | -23.78 | 2 | 21 | 118 | 138 | 3 guCGACCAACUUCCCCUGGUUu 5 | | ||| |||| ||||||| | 5 aaGGTGGGTGAA-AGGACCAAa 3 |
|  |  | NM_005409 | 162 | -23.78 | 2 | 21 | 158 | 178 | 3 guCGACCAACUUCCCCUGGUUu 5 | \| \|\|\| \|\|\|\| \|\|\|\|\|\|\| | 5 aaGGTGGGTGAA-AGGACCAAa 3 |
|  | DIEXF | NM_014388 | 162 | -26.83 | 2 | 21 | 4624 | 4644 | 3 guCGACCAACUUCCCCUGGUUu 5 | \|\|\|\|\| \|\| \|\|\|\|\|\|\|\| | 5 aaGCTGGGTG-TTGGGACCAAc 3 |
|  | FCGR3A | NM_000569 | 155 | -18.11 | 2 | 16 | 451 | 472 | 3 gucgaccAACUUCCCCUGGUUu 5 | \|\| \|: \|\|\|\|\|\|\|\| | 5 tagggaaTTCAGTGGGACCAAt 3 |
|  | PAG1 | NM_018440 | 154 | -26.75 | 2 | 20 | 7151 | 7177 | 3 gucGACCAACUUC-----CCCUGGUUu 5 | \|\|\|\| \|\|\|\| \|\|\|\|\|\|\|\| | 5 tctCTGGAAGAAGAGGCTGGGACCAAa 3 |
|  | POU3F3 | NM_006236 | 154 | -21.61 | 2 | 21 | 214 | 234 | 3 guCGACCAACUUCCCCUGGUUu 5 | \|\| \|\| \|\|\| \|\|\|\|\|\|\| | 5 acGC-GGGAGAAACGGACCAAg 3 |
|  | TTN | NM_133379 | 164 | -21.84 | 2 | 20 | 647 | 668 | 3 gucGACC-AACUUCCCCUGGUUu 5 | \|\|\|\| \|\| \|\| \|\|\|\|\|\|\|\| | 5 tcaCTGGCTTCAA-GGGACCAAt 3 |
| hsa-miR-193b-3p | BMP8A | NM_181809 | 151 | -18.48 | 2 | 17 | 3119 | 3142 | 3 ucgcccUGAAAC--UCCCGGUCAa 5 | |||||| ||||||| | 5 tcttaaACTTTGCCCAGGCCAGTc 3 |
|  | CEMIP | NM_001293298 | 151 | -18.36 | 2 | 20 | 837 | 858 | 3 ucgCCCUGAAACUCCCGGUCAa 5 | \|\| \| \|\|: \|\|\|\|\|\|\| | 5 agaGGTAAAATGGAGGCCAGTg 3 |
|  | EEF2 | NM_001961 | 155 | -29.1 | 2 | 20 | 220 | 241 | 3 ucgCCCUGAAACUCCCGGUCAa 5 | \|\|\|\|\|\|\| \|\|\|\|\|:\|\| | 5 gtaGGGACTTAATGGGCCGGTc 3 |
|  | KCNK10 | NM_138318 | 154 | -18.97 | 2 | 17 | 3049 | 3073 | 3 ucgcccUGAAA-CUC--CCGGUCAa 5 | \|:\|\|\| \|\|\| \|\|\|\|\|\|\| | 5 aaactaATTTTGGAGGAGGCCAGTt 3 |
|  | PPAN-P2RY11 | NM_001040664 | 150 | -20.29 | 2 | 19 | 510 | 531 | 3 ucgcCCUGAAACUCCCGGUCAa 5 | \|\|\| \|\| \|\|\|\|\|\|\| | 5 gcacGGAGCCAGATGGCCAGTc 3 |
|  |  | NM_001198690 | 159 | -22.63 | 2 | 21 | 494 | 516 | 3 ucGCCCU-GAAACUCCCGGUCAa 5 | :\|\| \| \| \|\|\|: \|\|\|\|\|\|\| | 5 tgTGGCAGCGTTGGTGGCCAGTg 3 |
|  |  | NM_001198690 | 150 | -20.29 | 2 | 19 | 1394 | 1415 | 3 ucgcCCUGAAACUCCCGGUCAa 5 | \|\|\| \|\| \|\|\|\|\|\|\| | 5 gcacGGAGCCAGATGGCCAGTc 3 |
|  | ZNF385B | NM_001113397 | 162 | -21.63 | 2 | 18 | 12 | 33 | 3 ucgccCUG-AAACUCCCGGUCAa 5 | \|\|\| \|\|\|\|\| \|\|\|\|\|\|\| | 5 cccaaGACGTTTGA-GGCCAGTa 3 |
| hsa-miR-194-3p | GPRC5B | NM_001304771 | 151 | -23.57 | 2 | 20 | 270 | 291 | 3 gucUAUUGUCGUCGGGGUGACc 5 | |||::| :|| |:||||| | 5 caaATAGTATTAGGCTCACTGg 3 |
|  | LEPROTL1 | NM_015344 | 150 | -25.22 | 2 | 15 | 1750 | 1771 | 3 gucuauugUCGUCGGGGUGACc 5 | \|\|\| :\|\|\|\|\|\|\| | 5 gtggctggAGCCTTCCCACTGg 3 |
|  | NME9 | NM_178130 | 153 | -23.54 | 2 | 18 | 512 | 533 | 3 gucuaUUGUCGUCGGGGUGACc 5 | \|:\|:\|\|\| \|\|:\|\|\|\|\| | 5 acctcAGCGGCACCCTCACTGc 3 |
|  | PAG1 | NM_018440 | 164 | -25.94 | 2 | 18 | 5991 | 6013 | 3 gucuaUUGUCG-UCGGGGUGACc 5 | \|:\|\|\|\| \| \|\|\|\|\|\|\|\| | 5 cctcaAGCAGCAACCCCCACTGt 3 |
|  | TBC1D7 | NM_001318806 | 150 | -18.63 | 2 | 19 | 1119 | 1140 | 3 gucuAUUGUCGUCGGGGUGACc 5 | \| :\| \| \|:\|\|\|\|\|\|\| | 5 gattTTGCTGATGTCCCACTGa 3 |
|  | VGLL3 | NM_001320494 | 150 | -21.51 | 2 | 20 | 29 | 52 | 3 gucUAUUGUCGU--CGGGGUGACc 5 | \|\|\|\| \|\| \| \|\|\|\|\|\|\| | 5 agcATAAATCCAAGGGCCCACTGg 3 |
|  | ZNF471 | NM_001321768 | 159 | -25.62 | 2 | 20 | 50 | 71 | 3 gucUAUUGUCGUCGGGGUGACc 5 | \|\| \| \|\| \|:\|\|\|\|\|\|\| | 5 ttaATCAGAGATGTCCCACTGg 3 |
| hsa-miR-194-5p | ARHGEF35 | NM_001003702 | 163 | -16.23 | 2 | 21 | 276 | 298 | 3 agGUGUACCUCA-ACGACAAUGu 5 | :|::|| | | ||||||||| | 5 aaTATGTGTATTCTGCTGTTACt 3 |
|  | DIP2C | NM_014974 | 158 | -18.87 | 2 | 20 | 443 | 461 | 3 aggUGUACCUCAACGACAAUGu 5 | \|\|\|\|\|\|\| \| \|\|\|\|\|\|\| | 5 atgACATGGA--T-CTGTTACt 3 |
|  |  | NM_014974 | 151 | -17.72 | 2 | 21 | 950 | 972 | 3 agGUGUACCUCA-ACGACAAUGu 5 | \|\|\|\|\| \| \| \|\|\|\|\|\|\|:\| | 5 ctCACATACACTCTGCTGTTGCc 3 |
|  | EOMES | NM_001278182 | 159 | -23.63 | 2 | 21 | 435 | 461 | 3 agGUGUACCUC-----AACGACAAUGu 5 | \|\| \| \|\|\|\| \|\|\|\|\|\|\|\|\|\| | 5 ttCAGAAGGAGACATTTTGCTGTTACa 3 |
|  | PEX2 | NM_000318 | 151 | -18.33 | 2 | 19 | 2627 | 2648 | 3 agguGUACCUC-AACGACAAUGu 5 | \|\| \|\|\|\| \| \|\|\|\|\|\|\| | 5 agtcCA-GGAGCTCACTGTTACt 3 |
|  | TMEM65 | NM_194291 | 156 | -18.16 | 2 | 20 | 1321 | 1340 | 3 aggUGUACCUCAACGACAAUGu 5 | :\|\|\|\| :\|\| \|\|\|\|\|\|\| | 5 ttaGCATGTGGT--CTGTTACa 3 |
| hsa-miR-33a-3p | ADAMDEC1 | NM_001145271 | 150 | -18.83 | 2 | 21 | 152 | 172 | 3 caCUACGUGACACCUUUGUAAc 5 | ||||:| | | ||:||||| | 5 caGATGTAAT-TAGAGACATTg 3 |
|  | CCR5 | NM_000579 | 150 | -21.05 | 2 | 21 | 1472 | 1495 | 3 caCUA-CGUGACAC-CUUUGUAAc 5 | \|\|\| \|\|\|\|\|\| \| \|\|:\|\|\|\| | 5 ggGATAGCACTGAGCAAAGCATTg 3 |
|  | CHCHD7 | NM_001011667 | 165 | -18.14 | 2 | 20 | 1129 | 1149 | 3 cacUACGUGACACCUUUGUAAc 5 | :\|\|:\|\|\| \|\| \|\|\|\|\|\|\| | 5 aatGTGTACT-TGAAAACATTg 3 |
|  |  | NM_001011668 | 165 | -18.14 | 2 | 20 | 998 | 1018 | 3 cacUACGUGACACCUUUGUAAc 5 | :\|\|:\|\|\| \|\| \|\|\|\|\|\|\| | 5 aatGTGTACT-TGAAAACATTg 3 |
|  |  | NM_001317858 | 165 | -18.14 | 2 | 20 | 1104 | 1124 | 3 cacUACGUGACACCUUUGUAAc 5 | :\|\|:\|\|\| \|\| \|\|\|\|\|\|\| | 5 aatGTGTACT-TGAAAACATTg 3 |
|  |  | NM_001317859 | 165 | -18.14 | 2 | 20 | 1090 | 1110 | 3 cacUACGUGACACCUUUGUAAc 5 | :\|\|:\|\|\| \|\| \|\|\|\|\|\|\| | 5 aatGTGTACT-TGAAAACATTg 3 |
|  | CXCL11 | NM_001302123 | 154 | -26.76 | 2 | 21 | 425 | 445 | 3 caCUACGUGACACCUUUGUAAc 5 | \|:\| :\|\| \|\|\|\|\|:\|\|\|\|\| | 5 gtGGT-TACGGTGGAGACATTg 3 |
|  |  | NM_005409 | 154 | -26.76 | 2 | 21 | 465 | 485 | 3 caCUACGUGACACCUUUGUAAc 5 | \|:\| :\|\| \|\|\|\|\|:\|\|\|\|\| | 5 gtGGT-TACGGTGGAGACATTg 3 |
|  | DIP2C | NM_014974 | 157 | -16.37 | 2 | 20 | 2094 | 2118 | 3 cacUACGUGAC--A-CCUUUGUAAc 5 | \| \|\|\|\|\|\| \| \|\|\|\|\|\|\|\| | 5 gccAGGCACTGACTATGAAACATTa 3 |
|  | ZMYND11 | NM_001202464 | 155 | -17.89 | 2 | 16 | 1728 | 1749 | 3 cacuacgUGACACCUUUGUAAc 5 | \|:\|\|\|\|\|\|\|\|:\|\|\| | 5 gcaagtaATTGTGGAAATATTt 3 |
| hsa-miR-3677-3p | ZNF471 | NM_001321768 | 152 | -27.5 | 2 | 21 | 243 | 264 | 3 ccGGCACCGGUCUCGGGUGCUc 5 | | |||| :: ||||||||: | 5 gaCAGTGGGTGCAGCCCACGGa 3 |
| hsa-miR-3917 | ATP6V1C1 | NM_001695 | 153 | -23.15 | 2 | 18 | 2775 | 2791 | 3 gggUGGACGAGUCAGGCUCg 5 | ||||| |||||||| | 5 ttaACCTG---AGTCCGAGa 3 |
|  | ZNF28 | NM_006969 | 158 | -21.76 | 2 | 15 | 1187 | 1206 | 3 ggguggACGAGUCAGGCUCg 5 | \| :\|\|\|\|\|\|\|\|\|\| | 5 gtaaggTCTTCAGTCCGAGa 3 |
|  | ZNF808 | NM_001039886 | 158 | -22.43 | 2 | 15 | 348 | 367 | 3 ggguggACGAGUCAGGCUCg 5 | \| :\|\|\|\|\|\|\|\|\|\| | 5 gcaaggTCTTCAGTCCGAGg 3 |
| hsa-miR-658 | APOE | NM_000041 | 158 | -27.89 | 2 | 21 | 33 | 56 | 3 ugguuGCCUGGAUGAAGGGAGGCGg 5 | || :||| | | ||||||| | 5 cacccCGTGCCT-CCTGCCTCCGCg 3 |
|  | DIEXF | NM_014388 | 159 | -27.9 | 2 | 24 | 2006 | 2030 | 3 ugGUUGCCUGGAUGAAGGGAGGCGg 5 | \|\|\| \|\|:: \| \| \|\|\|\|\|\|\| | 5 ttCAAGCGATTCTCGTGCCTCCGCc 3 |
|  | PNMA2 | NM_007257 | 152 | -28.56 | 2 | 24 | 1714 | 1742 | 3 ugGUU-GCCUGGA-UGAAG--GGAGGCGg 5 | \|\|\| \| \| \|\| \|\|\| \| \|\|\|\|\|\|\| | 5 tgCAATCTCAGCTCACTGCAACCTCCGCc 3 |
|  | RAB31 | NM_006868 | 170 | -28.3 | 2 | 24 | 777 | 802 | 3 ugGUUGCCUGGAUGAAG-GGAGGCGg 5 | \|\| \| \|\|\| \|:\| \|\| \|\|\|\|\|\|\| | 5 acCACCTGACATGCATCTCCTCCGCa 3 |
|  | ZNF28 | NM_006969 | 157 | -27.66 | 2 | 19 | 1611 | 1637 | 3 ugguugcCUGGAUGAAG--GGAGGCGg 5 | \|\|\|: \|\|\| \| \|\|\|\|\|\|\| | 5 tgatcttGACTCACTACAACCTCCGCc 3 |
| hsa-miR-708-5p | CEMIP | NM_001293298 | 154 | -22.72 | 2 | 17 | 1142 | 1163 | 3 gggucgaUCUAACAUUCGAGGAa 5 | ||| ||:||||||| | 5 agtccacAGA-AGTGAGCTCCTg 3 |
|  | CMTM7 | NM_138410 | 155 | -23.37 | 2 | 21 | 149 | 172 | 3 gggUCGAUCUA-ACAUUCGAGGAa 5 | \|\|\| \|\| \| \|:\|\|\|\|\|\|\| | 5 ggaAGCCAGCTCCCTGAGCTCCTg 3 |
|  |  | NM_181472 | 155 | -23.37 | 2 | 21 | 149 | 172 | 3 gggUCGAUCUA-ACAUUCGAGGAa 5 | \|\|\| \|\| \| \|:\|\|\|\|\|\|\| | 5 ggaAGCCAGCTCCCTGAGCTCCTg 3 |
|  | ELMSAN1 | NM_194278 | 162 | -21.09 | 2 | 19 | 3193 | 3215 | 3 gggucGAUCUAACAUUCGAGGAa 5 | \|\|\| \|\|\| :\|\|\|\|\|\|\| | 5 tccccCTATATTCAGAGCTCCTt 3 |
|  | GATAD2B | NM_020699 | 150 | -19.75 | 2 | 21 | 4265 | 4286 | 3 gggUCGAUCUAACAUUCGAGGAa 5 | \|\| \| \|\| \|:\|\|\|\|\|\|\| | 5 ccgAGGT-GAGGCTGAGCTCCTg 3 |
|  | LGSN | NM_001143940 | 152 | -18.51 | 2 | 21 | 3892 | 3914 | 3 gggUCGAUCUAACAUUCGAGGAa 5 | \|\|\|\|\| \| \| \|\|\|\|\|:\|\| | 5 aaaAGCTATTTGGAAAGCTTCTt 3 |
|  |  | NM_016571 | 152 | -18.51 | 2 | 21 | 3410 | 3432 | 3 gggUCGAUCUAACAUUCGAGGAa 5 | \|\|\|\|\| \| \| \|\|\|\|\|:\|\| | 5 aaaAGCTATTTGGAAAGCTTCTt 3 |
|  | REEP1 | NM_001164730 | 150 | -19.29 | 2 | 19 | 841 | 863 | 3 gggucGAUCUAACAUUCGAGGAa 5 | \|\|:\|\|\|\| :\|\|\|\|:\|\| | 5 ggattCTGGATTAGGAGCTTCTg 3 |
|  |  | NM_001164732 | 150 | -19.29 | 2 | 19 | 780 | 802 | 3 gggucGAUCUAACAUUCGAGGAa 5 | \|\|:\|\|\|\| :\|\|\|\|:\|\| | 5 ggattCTGGATTAGGAGCTTCTg 3 |
